# Interleukin-6 as an Oral–Vascular Inflammatory Bridge: Integrating CXCL10 and Haptoglobin 2-2 into a Potential Model of Periodontitis-Associated Cardiovascular Risk

**DOI:** 10.3390/ijms27177666

**Published:** 2026-08-27

**Authors:** David J. Vigerust, Bradley F. Bale

**Affiliations:** 1 Texas Tech University Health Sciences Center, Woody L. Hunt School of Dental Medicine, El Paso, TX 79905, USA; 2 SimplyTest LLC., Huntsville, AL 35806, USA; 3 Vanderbilt University School of Medicine, Nashville, TN 37232, USA; 4 BaleDoneen Method, Spokane, WA 99202, USA; 5 Elson S. Floyd College of Medicine, Washington State University, Spokane, WA 99202, USA; 6 Tanner College of Dentistry, University of Pikeville, Pikeville, KY 41501, USA

**Keywords:** periodontitis, cardiovascular disease, interleukin-6, CXCL10, haptoglobin 2-2, endothelial dysfunction, oral–systemic health, inflammation, biomarkers

## Abstract

Periodontitis is a chronic dysbiotic inflammatory disease increasingly recognized as a contributor to systemic inflammatory burden and cardiovascular risk. Consensus and mechanistic literature support an association between periodontitis and atherosclerotic cardiovascular disease, while recent interventional evidence suggests that intensive periodontal treatment can improve endothelial function, reduce systemic inflammation and oxidative stress, and favorably influence the progression of carotid intima-media thickness. Among candidate mediators, interleukin-6 (IL-6) is especially compelling because it participates in local periodontal inflammation, hepatic acute-phase activation, endothelial dysfunction, oxidative stress, and the biology of atherosclerotic cardiovascular disease. CXCL10 has emerged as a relevant adjunctive chemokine because it is detectable in saliva, serum, gingival crevicular fluid, and inflamed periodontal tissues. In addition, it has also been implicated in atherosclerosis and adverse cardiac remodeling. Proteogenomic evidence suggests that CXCL10 may act as a potential downstream mediator of IL-6-associated signaling in atherosclerosis. Haptoglobin 2-2 (Hp2-2), while not itself a primary cytokine driver, may function as a host-susceptibility modifier, as the Hp2-2 phenotype has been associated with impaired hemoglobin scavenging, HDL dysfunction, endothelial dysfunction, oxidative vulnerability, and higher cardiovascular risk, particularly in dysglycemic states. This review proposes an IL-6-centered model linking chronic periodontal inflammation to cardiovascular risk, with CXCL10 positioned as an inflammatory recruitment amplifier and Hp2-2 as a genotype-defined modifier of vascular susceptibility. The translational implications of this framework for precision oral–systemic risk assessment, integration of salivary and blood-based biomarkers, and future biomarker-guided intervention studies are discussed.

## 1. Introduction

The relationship between periodontal disease and cardiovascular disease has evolved from a largely epidemiologic association to a biologically plausible oral–systemic inflammatory model. Consensus reports and contemporary reviews support an association between periodontitis and atherosclerotic cardiovascular disease, while emphasizing that the causal architecture is complex and likely involves multiple overlapping pathways rather than a single linear mechanism [1,2,3]. Numerous publications provide mechanistic and interventional support for a contributory relationship between periodontitis and cardiovascular disease. Periodontal inflammation may contribute to systemic inflammatory burden and endothelial dysfunction, while periodontal treatment has been associated with improvements in several inflammatory and vascular surrogate measures [4,5,6,7,8]. In addition, high-risk periodontal pathogens may exacerbate proatherogenic inflammatory, oxidative, and metabolic pathways relevant to atherosclerosis [9]. Chronic inflammation from periodontal disease also influences systemic vascular biology through recurrent bacteremia, endotoxemia, innate immune activation, cytokine spillover, and endothelial dysfunction [1,10,11]. A major recent advance is the emergence of evidence from interventional studies. Orlandi and colleagues reported improved flow-mediated dilation, reductions in inflammatory and oxidative stress markers, and favorable effects on carotid intima-media thickness progression following intensive periodontal treatment [12,13,14]. These findings support the hypothesis that periodontal inflammation may contribute to atherogenic and vascular-remodeling processes, but they do not establish periodontitis as an independent causal cardiovascular risk factor [1,2,12].

**Figure 1:**
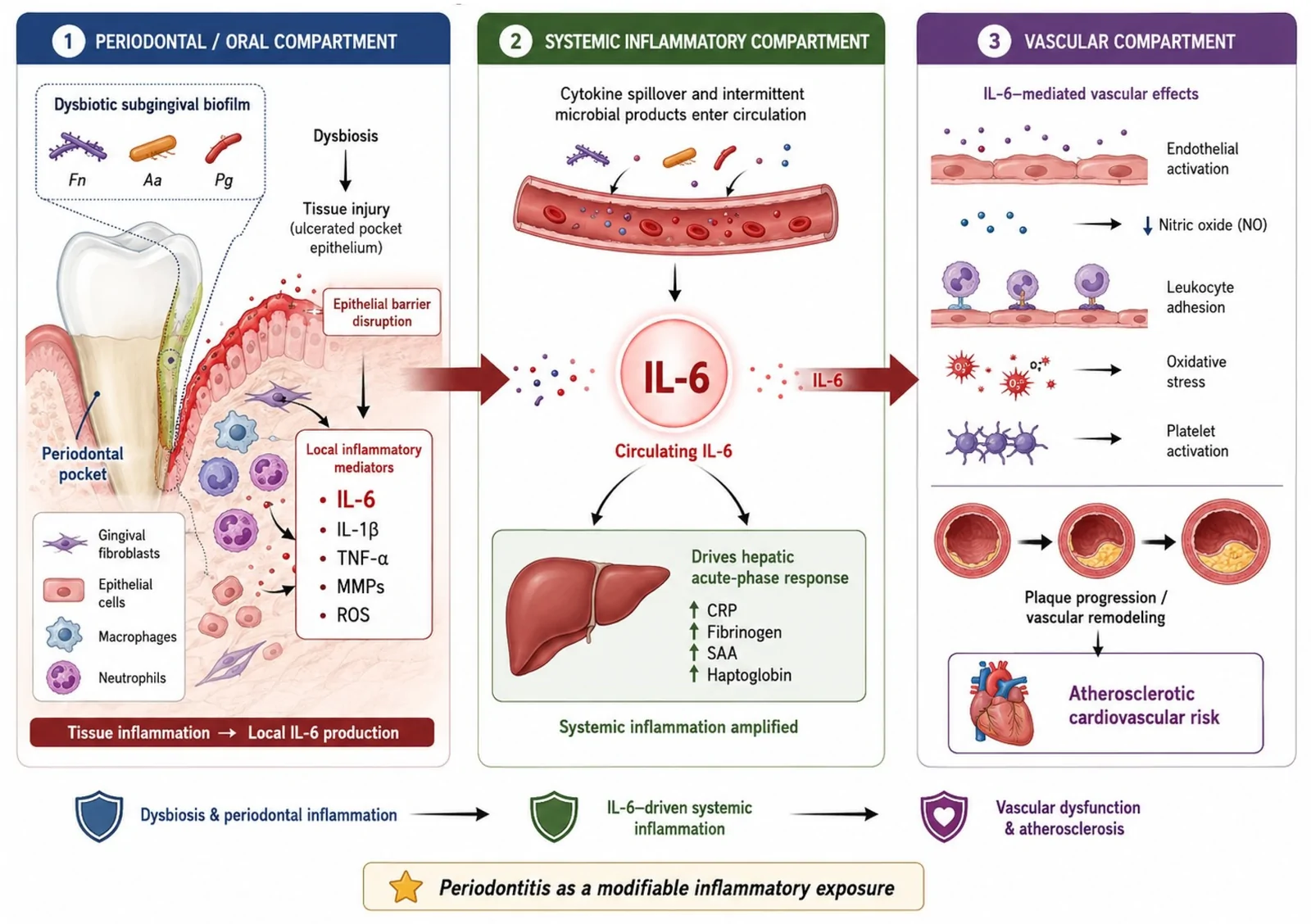
**Oral–Vascular IL-6 Axis Linking Periodontitis to Systemic Vascular Inflammation.** This model illustrates how chronic periodontitis may function as a sustained oral inflammatory exposure that contributes to systemic vascular risk. Dysbiotic subgingival biofilms containing organisms such as *Fusobacterium nucleatum*, *Aggregatibacter actinomycetemcomitans*, *Porphyromonas gingivalis*, and red/orange-complex communities promote epithelial barrier disruption, pocket ulceration, gingival bleeding, and activation of resident and recruited immune cells. Host tissues respond to microbial products and tissue injury by producing IL-6, IL-1β, TNF-α, matrix metalloproteinases, myeloperoxidase, and reactive oxygen species. Systemically, IL-6 promotes hepatic production of hsCRP, fibrinogen, serum amyloid A, complement components, and haptoglobin, thereby increasing inflammatory and prothrombotic burden. These signals converge on the vascular endothelium and contribute to vascular remodeling and atherosclerotic cardiovascular risk. The model positions IL-6 as a candidate integrative mediator within a broader oral–vascular inflammatory network rather than as a single causal pathway [1,2,3,23,26,27,28,29,30,31,34,36,37].

Two additional factors enrich this framework. First, C-X-C motif chemokine ligand 10 (CXCL10) is an IFN-γ–inducible chemokine that signals primarily through CXCR3, recruiting activated T cells and other inflammatory leukocytes into the vascular wall. Persistent CXCL10/CXCR3 signaling can amplify plaque inflammation and is associated with a more vulnerable plaque phenotype characterized by increased macrophages and reduced smooth muscle and collagen—suggesting roles not only in plaque development but also in plaque destabilization and cardiovascular events [15,16]. Increasingly, CXCL10 has been shown to be relevant to both periodontal inflammation and cardiovascular disease and may act downstream of IL-6-associated signaling in atherosclerosis [17,18,19,20,21,22,23,24,25]. Second, haptoglobin 2-2 (Hp2-2) is an acute-phase haptoglobin isotype that has been hypothesized to increase susceptibility to atherogenic oxidative injury [26,27,28,29]. Impaired hemoglobin handling associated with Hp2-2 may increase oxidative stress, lipoprotein oxidation, endothelial dysfunction, and impairment of reverse cholesterol transport, which can favor macrophage foam cell formation and plaque progression; the association appears particularly strong in diabetes and other high oxidative stress states and may influence how chronic inflammatory and oxidative burden translates into vascular injury, especially in patients with diabetes, dysglycemia, or increased oxidative stress [27,30,31,32,33,34,35]. The proposed model therefore integrates CXCL10 as a potential inflammatory-recruitment mediator and Hp2-2 as a host-susceptibility modifier within an IL-6-centered oral–vascular framework. IL-6 biology is well established in periodontitis and CVD; the intersection of CXCL10 with periodontal and systemic vascular inflammation is emerging, and we hypothesize that Hp2-2 may modify the vascular impact of oral inflammation. An overview of this proposed oral–vascular inflammatory framework is shown in Figure 1.

## 2. IL-6 Biology in Periodontitis

IL-6 is a central cytokine in periodontal inflammation because it is produced by multiple cell populations within the periodontal lesion and contributes to both local tissue destruction and systemic inflammatory signaling. In periodontal tissues, IL-6 can be produced by macrophages, monocytes, gingival fibroblasts, periodontal ligament fibroblasts, epithelial cells, lymphocytes, endothelial cells, osteoblast-lineage cells, and other stromal and immune cells exposed to bacterial products, inflammatory cytokines, oxidative stress, and tissue-injury signals [36,37,38,39,40,41]. Gingival fibroblasts are especially important because they are abundant resident stromal cells in gingival connective tissue and can respond directly to periodontal pathogens and inflammatory cytokines by producing IL-6, interleukin-8 (IL-8/CXCL8), prostaglandins, matrix-remodeling mediators, and other inflammatory signals [39,40].

Bartold and Haynes demonstrated that human gingival fibroblasts can synthesize IL-6, establishing them as active participants in periodontal cytokine biology rather than passive structural cells [39]. Takada and colleagues showed that periodontopathic bacterial lipopolysaccharides can induce interleukin-1 (IL-1) and IL-6 in human gingival fibroblasts, while Kent and colleagues later confirmed that lipopolysaccharide and inflammatory cytokines regulate IL-6 production by human gingival fibroblasts [40]. Patil, Rossa, and Kirkwood further demonstrated that *Aggregatibacter actinomycetemcomitans* lipopolysaccharide induces IL-6 expression through multiple mitogen-activated protein kinase pathways in periodontal ligament fibroblasts, implicating p38, extracellular signal-regulated kinase (ERK), and c-Jun N-terminal kinase (JNK) signaling in the fibroblast response to this pathogen [41].

In periodontitis, IL-6 contributes to inflammatory amplification by integrating microbial stimulation with host-response pathways, including Nuclear Factor of kappa-light-chain-enhancer of activated B cells (NF-κB), mitogen-activated protein kinase (MAPK), JAK/STAT, prostaglandin signaling, and osteoclastogenic pathways [36,37,38,40,41]. Periodontal pathogens and their virulence factors activate epithelial cells, fibroblasts, macrophages, and dendritic cells via pattern-recognition receptors and other cell-surface signaling systems, triggering coordinated production of interleukin-1 (IL-1), tumor necrosis factor (TNF-α), IL-6, interleukin-17 (IL-17), C-X-C motif chemokine ligand 8 (CXCL8)/interleukin-8 (IL-8), matrix metalloproteinases (MMPs), and reactive oxygen species (ROS) [36,37,38]. IL-6 then acts as both an effector and an amplifier by promoting leukocyte recruitment, B- and T-cell responses, Th17-associated inflammation, osteoclastogenesis, and local tissue-destructive programs that contribute to connective tissue degradation and alveolar bone loss [36,37,38].

Not all oral pathogens induce IL-6 to the same degree or through the same cell types. Rossano and colleagues showed that human monocytes and gingival fibroblasts release TNF-α, IL-1α, and IL-6 in response to particulate and soluble fractions of *Fusobacterium nucleatum* and related periodontopathogenic bacterial components [42]. Kang and colleagues showed that persistent exposure to *F. nucleatum* can trigger inflammatory changes in gingiva-derived mesenchymal stem cells and suppress their osteogenic differentiation capacity, consistent with the broader concept that *F. nucleatum* is a potent inducer of inflammatory signaling in periodontal tissues [43,44,45,46].

The inflammatory potency of *F. nucleatum* is particularly important because it occupies a strategic position in polymicrobial biofilm development. *F. nucleatum* can coaggregate with early and late colonizers and facilitate biofilm maturation, thereby helping to organize the microbial ecology in which red-complex organisms, orange-complex organisms, and other dysbiotic taxa interact [44,45,46]. Therefore, *F. nucleatum* may influence IL-6 biology directly by stimulating epithelial, fibroblast, and immune-cell systems and indirectly by promoting polymicrobial communities that amplify host inflammatory signaling [42,43,45,46,47].

*Porphyromonas gingivalis* also substantially contributes to IL-6 induction, but its immune effects are often more modulatory and cell-context-dependent than those of *F. nucleatum. P. gingivalis* expresses multiple virulence factors, including gingipains, atypical lipopolysaccharide structures, fimbriae, outer-membrane vesicles, and complement-modulating factors, allowing it to both stimulate and subvert host immune responses [48,49,50]. Lourbakos and colleagues showed that the arginine-specific protease from *P. gingivalis* activates protease-activated receptors and stimulates IL-6 secretion in oral epithelial cells, providing a direct mechanism by which gingipain activity may promote periodontal inflammatory events at the epithelial barrier [49]. Groeger and Meyle demonstrated that *P. gingivalis* can activate NF-κB and MAPK pathways in oral epithelial cells, while broader reviews emphasize that *P. gingivalis* can sustain inflammation and modulate host immune clearance [48].

These pathogen-specific observations argue against viewing IL-6 as the product of a single organism. Instead, IL-6 is best understood as a host-response output of dysbiosis. Organisms such as *F. nucleatum*, *A. actinomycetemcomitans*, and *P. gingivalis* may be among the strongest or most biologically relevant IL-6-inducing taxa, but the total IL-6 signal reflects the combined influence of microbial burden, virulence-factor expression, biofilm architecture, epithelial barrier disruption, neutrophil activity, macrophage-fibroblast crosstalk, and host susceptibility [36,37,38,39,40,41,42,45,46,47,48,49,50].

Clinical studies consistently show that IL-6 is elevated in periodontitis across saliva, gingival crevicular fluid (GCF), gingival tissue, and, more variably, serum and plasma, supporting its role as both a local periodontal inflammatory mediator and a potential marker of systemic inflammatory spillover. Salivary and GCF IL-6 generally increase with disease severity and can decline after periodontal therapy, while tissue studies confirm increased local IL-6 expression within diseased gingiva [37,51,52].

## 3. IL-6 in Cardiovascular Disease

In cardiovascular medicine, IL-6 has emerged as one of the most important inflammatory mediators of residual cardiovascular risk, particularly in patients whose LDL cholesterol, blood pressure, and other conventional risk factors are already being aggressively treated. Residual inflammatory risk refers to the persistent inflammatory burden that remains despite contemporary lipid-lowering and guideline-directed cardiovascular therapy. Clinically, this risk is often assessed downstream using hsCRP, but mechanistically, IL-6 is increasingly viewed as a more proximal component of the inflammatory cascade linking innate immune activation to vascular injury, plaque progression, thrombosis, and recurrent cardiovascular events [24,25,53,54,55,56,57,58,59,60].

Mechanistically, IL-6 occupies a central position between upstream innate immune activation and downstream hepatic, endothelial, and vascular responses. It is induced by IL-1β, TNF-α, microbial products, oxidized lipids, cellular stress, inflammasome activation, and tissue injury. Once produced, IL-6 signals through two complementary pathways. Classical signaling occurs when IL-6 binds the membrane-bound IL-6 receptor (IL-6R) on selected cells and recruits gp130, generally supporting regulated immune, regenerative, and acute-phase responses. Trans-signaling occurs when IL-6 binds soluble IL-6R, and the complex activates gp130 on a much broader range of cells, including endothelial and stromal cells, thereby extending and amplifying inflammation and making this pathway highly relevant to chronic inflammatory conditions such as periodontitis and atherosclerosis [37,53,54,55,56,57,61,62]. Through these pathways, IL-6 promotes endothelial activation, monocyte recruitment, macrophage polarization, vascular oxidative stress, smooth muscle cell migration, matrix remodeling, thrombosis-linked signaling, and amplification of the hepatic acute-phase response [24,25,53,54,55,56,57,58,59].

Genetic evidence strengthens the inference that IL-6 signaling is mechanistically involved in vascular disease. Mendelian randomization studies using variants in or near the IL-6R locus have shown that genetically downregulated IL-6 receptor signaling is associated with lower lifetime risk of coronary heart disease and other vascular outcomes [55,56]. Georgakis and colleagues reported that genetically downregulated IL-6 signaling was associated with reduced ischemic stroke risk and other cardiovascular outcomes, supporting the view that IL-6 signaling is mechanistically relevant rather than merely correlated with vascular disease [57]. More recent genetic perturbation data focused on IL-6 itself have reported that genetically downregulated IL-6 signaling is associated with lower lifetime risks of coronary artery disease, peripheral artery disease, and ischemic atherosclerotic stroke, further strengthening the inference that the IL-6 pathway is relevant in human atherosclerotic disease [24].

IL-6 is also intimately connected to acute-phase biology. Classic hepatocyte studies demonstrated that IL-6 is a major regulator of acute-phase protein synthesis in adult human hepatocytes [58]. Acute-phase proteins induced directly or indirectly by IL-6 include C-reactive protein, fibrinogen, serum amyloid A, complement components, and haptoglobin [25,58,59]. These proteins are not merely laboratory markers of inflammation; they influence leukocyte recruitment, endothelial activation, coagulation, oxidative stress, iron and heme handling, and immune amplification [24,25,53,54,55,56,57,58,59].

The CANTOS trial and related analyses provided clinical proof of concept that suppressing inflammation can reduce cardiovascular events without lowering LDL cholesterol. Although CANTOS targeted IL-1β rather than IL-6 directly, the IL-1β-IL-6-hsCRP cascade is biologically connected, and the magnitude of clinical benefit was closely related to downstream inflammatory suppression [54]. This strengthens the rationale for viewing IL-6 not only as a biomarker but also as a pathway that may help explain residual cardiovascular risk after conventional risk factors have been treated [24,25,53,54,55,56,57,58,59].

## 4. The Oral–Vascular IL-6 Axis

In this context, the oral cavity represents a uniquely accessible inflammatory compartment. Unlike many internal sources of chronic inflammation, periodontal inflammation can be directly visualized, measured, sampled, biologically characterized, and treated. Salivary and oral-fluid measurements of periodontal pathogens and host-response markers provide an accessible investigative approach for characterizing local inflammatory activity. Whether such profiles can prospectively identify individuals at increased vascular risk remains to be established [1,2,3,5,12,14,17,36].

The biological link between periodontal and vascular inflammation is unlikely to rely on a single route of dissemination. Rather, the periodontal lesion may influence systemic IL-6 biology through several complementary mechanisms. Ulceration of the periodontal pocket epithelium creates a chronically inflamed interface through which bacteria, bacterial products, danger-associated molecular patterns, and locally generated inflammatory mediators can enter the systemic circulation. Repeated transient bacteremia and exposure to microbial products, such as lipopolysaccharide, may activate circulating monocytes, neutrophils, endothelial cells, and hepatic innate immune pathways, thereby stimulating systemic cytokine production independently of, and in addition to, cytokines released directly from periodontal tissues. Thus, the oral–vascular IL-6 axis should not be interpreted as requiring that all circulating IL-6 originate within the gingiva. Rather, periodontal inflammation may serve as a persistent upstream stimulus that amplifies systemic IL-6 signaling through both direct and indirect pathways [1,2,3,53].

This distinction is important because IL-6 concentrations measured in serum or plasma reflect an integrated systemic signal rather than a tissue-specific fingerprint. Circulating IL-6 may originate from leukocytes, vascular cells, adipose tissue, skeletal muscle, liver-associated inflammatory pathways, or other inflammatory compartments. Periodontitis may therefore contribute to the systemic IL-6 burden by increasing local cytokine production and by generating microbial and inflammatory stimuli that induce IL-6 production at distant sites. The hypothesis proposed here is therefore one of periodontal contribution to systemic IL-6 signaling, rather than an exclusive periodontal origin of circulating IL-6.

Once systemic IL-6 signaling is increased, established hepatic and vascular pathways may translate this signal into systemic inflammatory and vascular effects, as outlined above. In the proposed framework, periodontal inflammation would therefore contribute to an already multifactorial systemic inflammatory environment rather than act as an isolated cause of vascular disease.

A useful conceptual distinction is therefore between three interconnected compartments. The periodontal compartment contains the initiating dysbiotic biofilm, epithelial barrier disruption, inflammatory cell infiltration, and local IL-6 production. The systemic compartment integrates cytokines, microbial products, activated leukocytes, hepatic acute-phase proteins, and metabolic inflammatory signals. The vascular compartment is the downstream target where endothelial activation, oxidative stress, leukocyte recruitment, lipid modification, and plaque-associated inflammation occur. IL-6 is particularly attractive as a candidate bridge among these compartments because it participates in the biology of each.

Periodontal intervention studies provide indirect support for this compartmental model. Periodontal therapy has repeatedly been associated with reductions in systemic inflammatory burden and improvements in measures of endothelial function, although effects on individual cytokines, including circulating IL-6, are heterogeneous across studies and patient populations. A recent meta-analysis found improvements in endothelial function and reductions in C-reactive protein after periodontal therapy, while the evidence for a reduction in circulating IL-6 was directionally favorable but of lower certainty. These findings are consistent with the hypothesis that reducing periodontal inflammatory input can alter systemic and vascular inflammatory biology, but they do not establish that IL-6 is the sole mediator or that periodontal therapy reduces major adverse cardiovascular events.

The oral–vascular IL-6 model should also be distinguished from parallel mechanisms that may act independently of IL-6. Periodontal bacteria or their products can potentially interact directly with endothelial and immune cells, while IL-1β, TNF-α, prostaglandins, neutrophil-derived mediators, oxidative stress pathways, and trained innate immune responses may contribute additional systemic effects. IL-6 is therefore best positioned as a candidate integrative mediator within a broader network, rather than as the exclusive molecular conduit between periodontitis and cardiovascular disease.

From a translational perspective, the strongest test of the oral–vascular IL-6 axis will require temporally matched measurements across biological compartments. Saliva and gingival crevicular fluid can characterize local periodontal inflammatory activity; serum or plasma IL-6, hsCRP, GlycA, fibrinogen, and related markers can characterize systemic inflammatory responses; and flow-mediated dilation, vascular imaging, endothelial biomarkers, or cardiovascular outcomes can assess downstream vascular consequences. Demonstration that successful periodontal treatment produces coordinated changes across these compartments and that the magnitude of these changes tracks with baseline periodontal inflammatory burden would provide substantially stronger evidence for an oral–vascular inflammatory axis than cross-sectional biomarker associations alone. Accordingly, the proposed model does not require circulating IL-6 to originate directly from periodontal tissues; rather, periodontitis may amplify systemic IL-6 signaling through both local cytokine release and secondary systemic immune activation.

## 5. CXCL10 as an Emerging Chemokine Link

CXCL10, also known as interferon-γ-inducible protein 10 or IP-10, is a CXC chemokine that participates in Th1-associated immune responses by recruiting activated T cells, natural killer cells, monocytes, and other CXCR3-expressing inflammatory cells into sites of tissue injury [17,18,20,22]. Unlike IL-6, which is broadly induced by innate immune activation, microbial products, and tissue injury, CXCL10 more strongly reflects interferon-γ-rich and Th1-amplifying inflammatory environments [17,18,19,20,22]. This distinction is important because it positions CXCL10 as a complementary marker to IL-6: IL-6 captures acute-phase and innate inflammatory burden, while CXCL10 may capture the recruitment and persistence of a more lymphocyte-directed inflammatory program [17,18,19,20,21,22].

In periodontal disease, CXCL10 has been detected in gingival crevicular fluid, saliva, serum, and inflamed gingival tissues and has been proposed as a candidate biomarker of periodontal inflammatory activity [17,18,20]. Aldahlawi and colleagues evaluated CXCL10 concentrations in saliva, serum, and gingival crevicular fluid and reported higher CXCL10 levels in subjects with chronic periodontitis compared with periodontally healthy controls, supporting its relevance as a measurable oral–systemic inflammatory mediator [17]. Sahingur and colleagues reviewed chemokine function in periodontal disease and emphasized that chemokines participate in leukocyte trafficking, inflammatory cell retention, and immune polarization within periodontal lesions [18,20].

The regulatory biology of CXCL10 differs from that of IL-6. Hosokawa and colleagues showed that cytokines differentially regulate CXCL10 production in interferon-γ or TNF-α-stimulated human gingival fibroblasts, indicating that gingival stromal cells can be a meaningful source of CXCL10 when exposed to the cytokine milieu typical of chronic inflammation [19]. This is biologically important because gingival fibroblasts are strategically positioned within the periodontal connective tissue and can integrate bacterial, cytokine, and mechanical signals. When fibroblasts produce CXCL10, they may help convert a local stromal inflammatory response into a chemokine gradient that recruits additional CXCR3-positive immune cells, thereby sustaining chronicity [18,19].

Importantly, not all periodontal pathogens are strong inducers of CXCL10. Jauregui and colleagues reported that *P. gingivalis* did not induce the T-cell chemokine CXCL10 from neutrophils, peripheral blood mononuclear cells, or macrophages and could suppress T-cell chemokine responses, highlighting an immune-subversive strategy rather than straightforward chemokine activation [21]. This observation aligns with the broader biology of *P. gingivalis* as a keystone pathogen capable of manipulating host immune responses, disrupting leukocyte recruitment, and promoting dysbiosis while preserving destructive inflammation [21,49,50].

CXCL10 is also increasingly relevant to cardiovascular disease. Proteogenomic integration by Prapiadou and colleagues identified CXCL10 as a potential downstream mediator of IL-6-associated signaling using proteogenomic causal-inference approaches [22]. This finding pertains to vascular IL-6 biology and does not establish a periodontal IL-6→CXCL10→atherosclerosis pathway. It nevertheless provides a rationale for investigating whether CXCL10 participates in the proposed oral–vascular inflammatory network. Within the proposed framework, CXCL10 may represent one mechanism through which an IL-6-associated inflammatory environment is linked to sustained leukocyte recruitment and plaque-associated inflammation [17,18,19,20,21,22].

Emerging cardiovascular research also suggests that CXCL10 may influence myocardial remodeling. Souza-Neto and colleagues reported that CXCL10 regulates pressure-overload-induced remodeling in heart failure, suggesting that its biology may extend beyond plaque-associated vascular inflammation to encompass immune-cell recruitment, fibrosis-linked remodeling, and myocardial structural adaptation [23]. Although this area remains less mature than the IL-6 cardiovascular literature, it supports the concept that CXCL10 may participate in broader cardiovascular inflammatory remodeling rather than function solely as a local periodontal chemokine [22,23].

**Figure 2:**
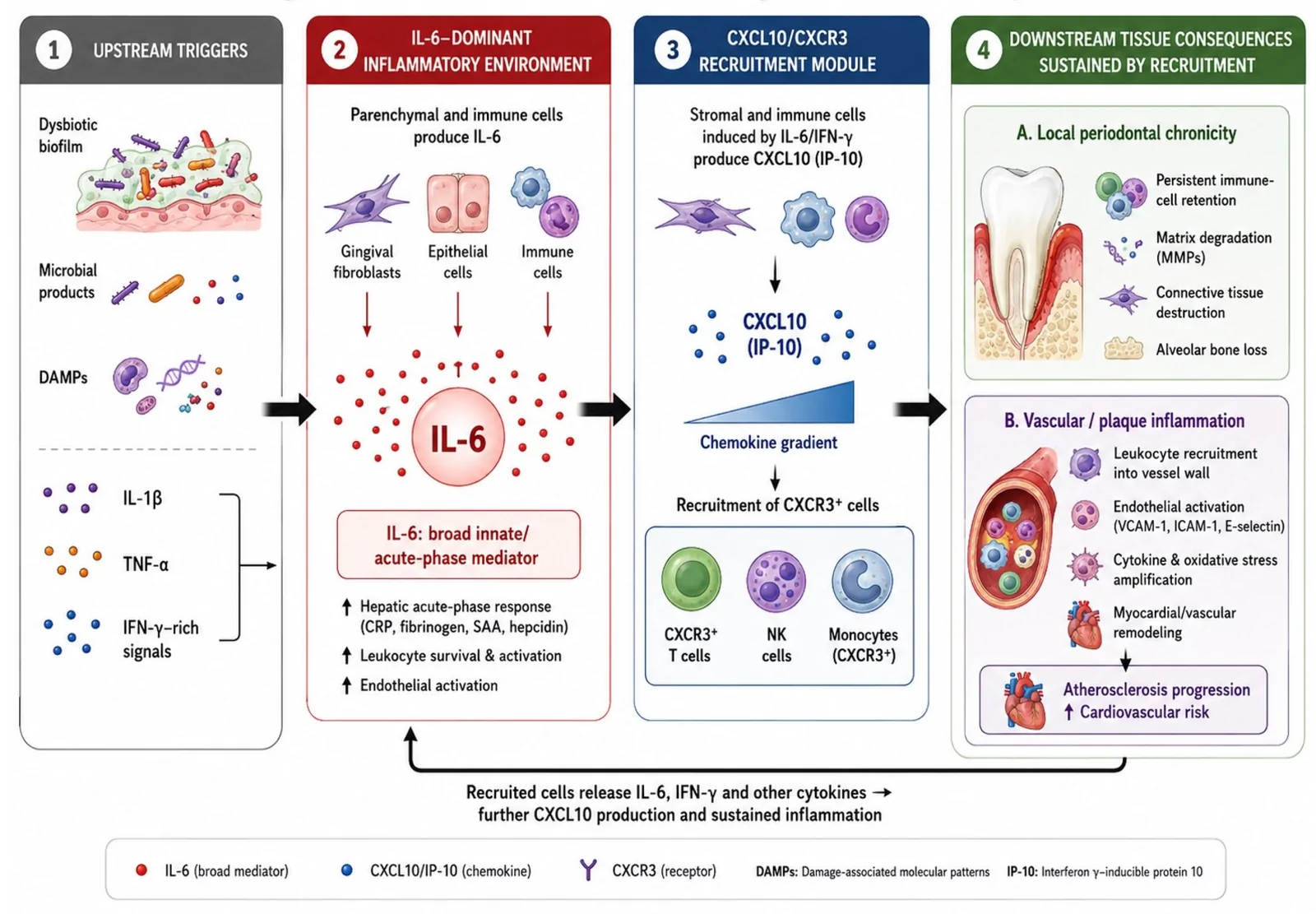
**IL-6/CXCL10 Inflammatory Recruitment Amplifier Model in Oral and Vascular Tissues.** This schematic depicts CXCL10/IP-10 as a chemokine amplifier that may extend and sustain IL-6-associated inflammation. Microbial products, danger-associated signals, IL-1β, TNF-α, and innate immune activation promote an IL-6-dominant inflammatory environment within periodontal tissues. In parallel, interferon-γ- and TNF-α-rich signaling stimulates gingival fibroblasts, stromal cells, and immune cells to produce CXCL10. CXCL10 establishes a chemokine gradient that recruits CXCR3-expressing activated T cells, natural killer cells, monocytes, and other inflammatory cells to sites of tissue injury. This recruitment may exacerbate chronic periodontal inflammation by increasing immune cell retention, cytokine production, stromal activation, and tissue-destructive signaling. In the vascular compartment, a similar IL-6/CXCL10-linked program may contribute to leukocyte recruitment into the vessel wall, plaque inflammation, endothelial activation, and potentially myocardial or vascular remodeling [5,14,15,16,17,18,19,37,39].

Accordingly, CXCL10 is best understood as a potential amplifier of inflammation within the oral–cardiovascular axis. It is less well established than IL-6 and should not be overinterpreted as a standalone causal biomarker in oral–systemic disease. However, its biology is sufficiently well-developed to justify prospective evaluation in exploratory biomarker studies, particularly when combined with IL-6, conventional periodontal measures, and established systemic inflammatory markers [17,18,19,20,21,22,23]. A testable question is whether combined measurements of IL-6 and CXCL10 can identify biologically distinct inflammatory phenotypes more effectively than either marker alone [17,18,19,20,21,22,23]. The hypothesized IL-6/CXCL10 inflammatory recruitment amplifier model is illustrated in Figure 2.

## 6. Haptoglobin 2-2 as a Host Susceptibility Modifier

Haptoglobin is an IL-6-responsive acute-phase protein involved in hemoglobin scavenging, iron handling, antioxidant defense, and modulation of inflammatory injury [30]. Its core protective function is to bind cell-free hemoglobin released during hemolysis or tissue injury, thereby reducing hemoglobin-driven oxidative stress, heme toxicity, nitric oxide scavenging, endothelial dysfunction, and lipid oxidation [27,30,31,32,33,34]. In this sense, haptoglobin is part of the host defense response to inflammatory and oxidative stress. However, the clinical relevance of haptoglobin is strongly influenced by genetic variation at the HP locus, which gives rise to Hp1-1, Hp2-1, and Hp2-2 phenotypes [30].

Mechanistically, the Hp2-2 phenotype appears to handle free hemoglobin and oxidative stress less efficiently than Hp1-containing phenotypes, especially under diabetic or hyperglycemic conditions [27,31,32,33,34,35]. Hp2-2 forms larger polymers that may have reduced antioxidant and hemoglobin-clearance efficiency compared with Hp1-1, leading to greater hemoglobin-associated oxidative injury [27,30,31,32,33,34]. In diabetes, increased glycation, oxidative stress, endothelial vulnerability, and lipoprotein dysfunction may magnify the adverse consequences of Hp2-2 biology [31,32,33,34]. Thus, Hp2-2 is best understood as a susceptibility phenotype that modifies the vascular consequences of inflammatory and oxidative stress rather than as a primary inflammatory initiator [27,31,32,33,34,35]. Although Hp2-2 has not yet been validated as a modifier of periodontitis-cardiovascular disease (CVD) risk, there is reason to hypothesize that it may serve as a susceptibility factor for CVD risk.

Cardiovascular literature supports this interpretation. Mewborn and colleagues reported that Hp2-2 was associated with substantially higher odds of coronary artery disease (CAD) than Hp1-1 in a case–control study of individuals with prediabetes [28]. Cahill and colleagues reported that haptoglobin genotype is a consistent marker of coronary heart disease risk among individuals with elevated glycosylated hemoglobin, suggesting that the Hp2-2 risk is particularly pronounced when glycemic burden is increased [31,32]. Related analyses found that the coronary heart disease risk associated with HbA1c of 6.5% or greater was especially pronounced among individuals with the Hp2-2 genotype, reinforcing the concept of a gene-metabolic interaction [31,32].

The HDL connection is central to the mechanistic model. Asleh and colleagues reported that the haptoglobin phenotype is associated with high-density lipoprotein-bound hemoglobin content and coronary endothelial dysfunction in patients with mild nonobstructive coronary artery disease [33,34]. In individuals with diabetes and Hp2-2, hemoglobin can become more closely associated with high-density lipoprotein (HDL), impairing HDL’s antioxidant function and potentially converting HDL from a protective lipoprotein into a less effective or even dysfunctional particle under oxidative stress [33,34]. This provides a plausible pathway by which Hp2-2 could amplify vascular injury in the presence of chronic inflammation: IL-6 and periodontal inflammation increase systemic acute-phase and oxidative burdens; Hp2-2 reduces the efficiency of hemoglobin-related antioxidant protection; HDL function is impaired; endothelial dysfunction and lipid oxidation intensify [28,31,32,33,34,63].

The oral literature on Hp2-2 remains limited but intriguing. Ebadian and colleagues examined haptoglobin gene polymorphisms in peri-implantitis and chronic periodontitis, while Kadkhodazadeh and colleagues explored associations between haptoglobin alleles, natural resistance-associated macrophage protein 1 alleles, and heme-consuming periodontal pathogens in chronic periodontitis and peri-implantitis [64]. These studies are insufficient to establish Hp2-2 as a direct determinant of periodontal disease severity, nor do they demonstrate that Hp2-2 directly links periodontitis to cardiovascular events. However, they suggest that haptoglobin biology is relevant to oral inflammatory ecosystems, particularly because periodontal pathogens interact with heme acquisition, bleeding, iron availability, oxidative stress, and host inflammatory responses [64].

**Figure 3:**
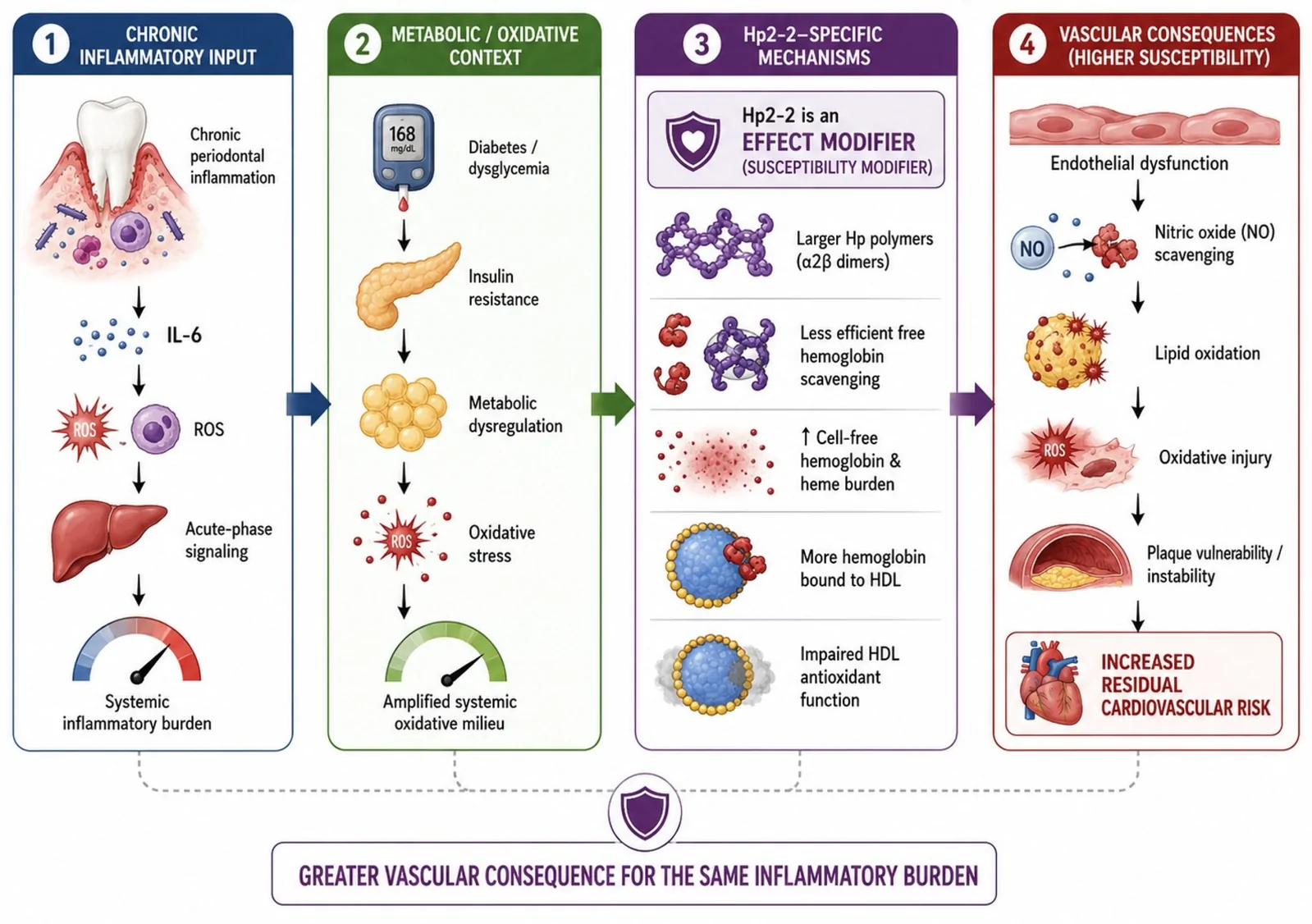
**Haptoglobin 2-2 as a Host Susceptibility Modifier of Oxidative and Endothelial Injury.** This model positions the haptoglobin 2-2 phenotype as a host factor that may increase vulnerability to the vascular consequences of chronic oral inflammation. Periodontal inflammation increases IL-6 levels, oxidative stress, acute-phase signaling, and the microbial inflammatory burden. In individuals with dysglycemia, diabetes, insulin resistance, or other metabolic stressors, oxidative and endothelial vulnerability may be further amplified. Hp2-2 is modeled as a susceptibility phenotype characterized by less efficient hemoglobin scavenging and reduced protection against hemoglobin-, heme-, and iron-driven oxidative injury compared with Hp1-containing phenotypes. Increased cell-free hemoglobin and HDL-bound hemoglobin may impair HDL antioxidant function, promote nitric oxide scavenging, enhance lipid oxidation, and worsen endothelial dysfunction [27,28,29,30,31,32,33,34,63].

In the present oral–vascular model, the strongest interpretation is that Hp2-2 modifies host vulnerability to the vascular consequences of chronic oral inflammation. Periodontitis can provide a persistent source of IL-6, microbial products, oxidative stress, and acute-phase signaling [1,2,3,37,38,39,41,42,49,50]. Hp2-2 may then reduce vascular resilience to this inflammatory burden by weakening hemoglobin scavenging, increasing HDL-bound hemoglobin, impairing HDL antioxidant function, and worsening endothelial dysfunction, especially in patients with diabetes, dysglycemia, insulin resistance, or other factors that amplify oxidative stress [28,31,32,63]. This interpretation avoids overstating the evidence while preserving the translational importance of Hp2-2 as a genotype-defined risk modifier. The hypothesized Hp2-2 susceptibility model is shown in Figure 3.

## 7. Feed-Forward and Feedback Mechanisms Linking IL-6, CXCL10, and Hp2-2

The proposed interaction among IL-6, CXCL10, and Hp2-2 should not be interpreted as a symmetric signaling triad. CXCL10 is regulated predominantly by IFN-γ/STAT1- and TNF-associated pathways, although proteogenomic evidence places it downstream of IL-6-associated signaling in vascular disease. It is therefore best regarded as a convergent inflammatory-recruitment mediator rather than an obligate direct IL-6 effector [17,18,19,20,21,22,23].

Hp2-2 occupies a different position in the model. Current evidence does not establish Hp2-2 as an upstream regulator of IL-6 or CXCL10; rather, the phenotype may increase susceptibility to oxidative and endothelial injury once inflammation is established. The testable hypothesis is therefore that a similar periodontal inflammatory burden may have greater vascular consequences in Hp2-2 individuals, particularly in the presence of dysglycemia [12,17,18,19,20,21,22,23,27,30,31,32,33,34,35,63,64,65,66].

## 8. Integrated Model, Clinical Context, and Translational Implications

Beyond periodontal debridement and local infection control, several adjunctive strategies may reduce the IL-6/CXCL10-associated inflammatory burden or mitigate the oxidative and vascular consequences of Hp2-2 susceptibility. These strategies act at different levels: some reduce upstream inflammatory stimuli, some blunt innate inflammatory signaling, and others improve the metabolic and oxidative environments that exacerbate cytokine-driven injury. The practical relevance of this framework is that oral–systemic inflammatory risk may be modifiable even without direct IL-6-targeted biologics [12].

The first and most defensible intervention remains control of the periodontal inflammatory source. Scaling and root planing, comprehensive periodontal therapy, biofilm disruption, management of deep pockets, improved home care, smoking cessation, glycemic optimization, and periodontal maintenance are the core interventions most directly linked to reducing periodontal inflammatory stimuli and local IL-6/CXCL10-associated activity [2,3,5,12,36,66]. If validated prospectively, biomarker-guided follow-up could help determine whether clinical periodontal improvement is accompanied by meaningful reductions in systemic inflammatory burden [5,12,14,17,66,67,68].

Therefore, the therapeutic hierarchy should prioritize control of the oral inflammatory source, optimization of cardiometabolic amplifiers such as smoking, dysglycemia, obesity, and hypertension, and coordinated medical management of systemic inflammatory risk when clinically indicated [3,5,12,36,54,66].

The proposed framework provides a rationale for investigating integrated microbial-host biomarker profiles rather than pathogen-only approaches. In future prospective studies, salivary or gingival crevicular fluid IL-6 and CXCL10 could be evaluated alongside microbial burden, clinical periodontal status, and established systemic inflammatory measures to determine whether a multi-marker approach provides incremental biological or prognostic information [2,3,12,17,18,19,20,21,22,23,27,30,31,32,33,34,35,36,63,64,65,66,67,68].

At present, IL-6, CXCL10, and Hp2-2 should not be regarded as established clinical biomarkers for routine periodontal or cardiovascular risk assessment. None is currently recommended as a replacement for conventional periodontal examination, radiographic assessment, or validated cardiovascular risk-estimation methods. The analytical feasibility of measuring these biomarkers should therefore not be interpreted as evidence of established clinical utility [69]. Their proposed integration into the present model is therefore investigational and hypothesis-generating, intended to identify testable biomarker combinations for prospective validation rather than to define a current clinical diagnostic algorithm [69].

## 9. Future Directions

Prospective, multicenter, interventional studies that track both biomarkers and cardiovascular events are clearly needed. Such studies remain scarce, in part because objective measurements of microbial and host-response biomarkers have not yet been routinely integrated into periodontal practice or longitudinal cardiovascular studies. Future studies in both medicine and dentistry should simultaneously evaluate periodontal status, oral microbial burden, IL-6, CXCL10, haptoglobin phenotype, metabolic status, endothelial function, vascular imaging, and cardiovascular outcomes in prospective cohorts [12,17,18,19,20,21,22,23,27,30,31,32,33,34,35,63,64,65,66,70].

A central translational question is whether the proposed biomarkers provide information beyond that provided by established cardiovascular risk assessment. Future studies should therefore evaluate periodontal inflammatory measures, IL-6, CXCL10, and haptoglobin phenotype alongside validated cardiovascular risk factors and risk-estimation approaches. Analyses should assess not only statistical association but also discrimination, calibration, risk reclassification, and clinical utility. Biomarkers that are associated with disease but do not improve risk estimation or management beyond established methods may have limited clinical value. Biomarker-stratified intervention studies are especially needed to determine whether baseline inflammatory phenotype modifies vascular response to periodontal therapy. The Orlandi trial provides an important template, but future work should incorporate deeper biomarker phenotyping and host-susceptibility markers to determine which patients experience the greatest systemic benefit from periodontal intervention [12].

A critical test of the proposed oral–vascular inflammatory model is not simply whether periodontal, inflammatory, and vascular biomarkers are correlated at a single time point, but whether modifying the oral inflammatory source elicits a coordinated biological response across compartments. Specifically, the model predicts that effective periodontal treatment would produce coordinated reductions in oral inflammatory burden and, in at least a subset of patients, corresponding changes in systemic inflammatory phenotype and vascular function. Importantly, the magnitude of this vascular response may vary by haptoglobin genotype.

A central falsifiable prediction of the model is that the vascular consequences of a given periodontal inflammatory burden will be greater in individuals with the Hp2-2 phenotype than in those with Hp1-containing phenotypes, particularly in the presence of dysglycemia or diabetes. This prediction provides a directly testable framework for prospective, biomarker-stratified intervention studies and would help determine whether Hp2-2 functions as a clinically meaningful effect modifier of the oral–vascular inflammatory relationship.

Standardization of biomarker collection and analysis will be essential before cross-study comparisons or clinical translation are possible. Salivary measurements may vary depending on whether collection is stimulated versus unstimulated, collection time, fasting status, recent food intake or oral hygiene, oral bleeding, flow rate, collection device, storage conditions, and freeze–thaw exposure. GCF measurements are also influenced by sampling site, fluid volume, pocket characteristics, and contamination, while serum and plasma values may differ depending on matrix and processing conditions. Analytical studies should also establish assay calibration, sensitivity, matrix effects, normalization procedures, and interlaboratory reproducibility. Future prospective studies should therefore employ standardized and temporally matched collection across oral and systemic compartments [3,12,17,18,19,20,21,22,23,27,30,31,32,33,34,35,36,63,64,65,66].

## 10. Limitations of the Proposed Model

Although substantial evidence independently links periodontitis to systemic inflammation, IL-6 signaling to atherosclerotic biology, CXCL10 to inflammatory leukocyte recruitment, and Hp2-2 to oxidative vascular susceptibility, direct evidence linking these components within a single periodontal-to-vascular causal pathway remains incomplete. Current biomarker studies cannot establish the periodontal origin of circulating IL-6; CXCL10 is regulated by multiple inflammatory pathways beyond IL-6; and Hp2-2 is best regarded as a context-dependent susceptibility modifier rather than a component of the cytokine signaling cascade. Shared cardiometabolic determinants, including smoking, obesity, diabetes, dyslipidemia, aging, and systemic inflammatory disease, may independently influence periodontal status, IL-6/CXCL10 concentrations, endothelial function, and cardiovascular risk. Moreover, periodontal intervention studies demonstrating improvements in systemic inflammation and vascular surrogate endpoints have not yet established reductions in major adverse cardiovascular events. The proposed IL-6–CXCL10–Hp2-2 framework should therefore be regarded as a biologically informed and testable integrative hypothesis that requires prospective, compartment-resolved biomarker studies and biomarker-stratified intervention trials. In addition, the incremental clinical value of IL-6, CXCL10, Hp2-2, or their combination beyond conventional periodontal assessment and established cardiovascular risk-estimation methods remains unknown.

## 11. Conclusions

IL-6 is among the most biologically plausible candidate mediators linking chronic periodontal inflammation with cardiovascular risk. It is produced within periodontal lesions, contributes to local tissue destruction, drives hepatic acute-phase responses, contributes to oxidative stress [71], and participates in vascular inflammatory signaling [5]. CXCL10 appears to be an important emerging chemokine amplifier within this axis, particularly because it is detectable in periodontal compartments and has been implicated as a potential downstream mediator of IL-6 signaling in atherosclerosis [17,18,19,20,21,22,23]. Hp2-2 is best understood as a host susceptibility modifier that may increase the vascular consequences of sustained inflammatory and oxidative burden, especially in dysglycemic or diabetic patients [27,30,31,32,33,34,35,63,64,65].

Together, these pathways support an expanded oral–systemic model in which periodontitis contributes not only to local tissue destruction but also to systemic vascular risk biology. The model remains translational and hypothesis-generating but is biologically coherent and directly testable. Prospective studies integrating periodontal status, microbial burden, IL-6, CXCL10, haptoglobin phenotype, metabolic status, established cardiovascular risk measures, and vascular outcomes are required to determine whether this framework provides clinically meaningful information beyond current periodontal and cardiovascular assessment methods.

## Data Availability

The original contributions presented in this study are included in the article. Further inquiries can be directed to the corresponding author.
